# Lignin-Based Hybrid Admixtures and their Role in Cement Composite Fabrication

**DOI:** 10.3390/molecules24193544

**Published:** 2019-09-30

**Authors:** Łukasz Klapiszewski, Izabela Klapiszewska, Agnieszka Ślosarczyk, Teofil Jesionowski

**Affiliations:** 1 Poznan University of Technology, Faculty of Chemical Technology, Institute of Chemical Technology and Engineering, Berdychowo 4, PL-60965 Poznan, Poland; teofil.jesionowski@put.poznan.pl; 2 Poznan University of Technology, Faculty of Civil and Environmental Engineering, Institute of Structural Engineering, Piotrowo 5, PL-60965 Poznan, Poland; izabela.klapiszewska@put.poznan.pl

**Keywords:** biopolymers, inorganic-organic hybrid materials, cement composites

## Abstract

In this study, a technology for obtaining functional inorganic-organic hybrid materials was designed using waste polymers of natural origin, i.e., kraft lignin and magnesium lignosulfonate, and alumina as an inorganic component. Al_2_O_3_-lignin and Al_2_O_3_-lignosulfonate systems were prepared by a mechanical method using a mortar grinder and a planetary ball mill, which made it possible to obtain products of adequate homogeneity in an efficient manner. This was confirmed by the use of Fourier transform infrared spectroscopy and thermogravimetric analysis. In the next step, the developed hybrid materials were used as functional admixtures in cement mixtures, thus contributing to the formation of a modern, sustainable building material. How the original components and hybrid materials affected the mechanical properties of the resulting mortars was investigated. The admixture of biopolymers, especially lignin, led to cement composites characterized by greater plasticity, while alumina improved their strength properties. It was confirmed that the system containing 0.5 wt.% of alumina-lignin material is the most suitable for application as a cement mortar admixture.

## 1. Introduction

Next to cellulose, lignin is the second most abundant plant resource. It is characterized by an irregular and branched chemical structure, resulting from the condensation of three phenylpropanoid units [1]. A renewable, non-toxic biopolymer, it is commonly found in the central part of the cell wall of plants, where it confers rigidity, regulates the flow of minerals and protects the cell against biochemical hazards [2]. Due to its rich chemical structure, lignin has found a number of applications. It can be used as a source of highly functional chemicals, adsorbents, flocculants and scale inhibitors [3]. There are also numerous literature reports regarding the use of lignin as a filler for polymers [4,5,6] or a component of abrasive tools [7,8]. The variety of oxygen-containing functional groups enables the use of lignin-based materials for removal of various contaminants from water, mainly environmentally hazardous metal ions [9,10,11]. In addition to these applications, the biopolymer is also used in construction, mining, metallurgy and the oil industry. Approximately half of the lignin-derived products manufactured worldwide are used for production of cement and concrete. Additionally, these materials are used as additives (0.5–2.0%) for production of bricks and ceramics, as components of plasterboard (0.1–0.3%), or for reducing the dust content in road construction [12]. The continuous expansion of potential applications contributes to the development of methods for binding chlorinated organic wastes from lignin hydrolysis plants in order to obtain modifying additives in road building [12]. The biopolymer is also used as a binder for glass wool insulation for buildings [13]. Haro et al. [14] successfully used lignin in renewable coatings with anti-corrosive properties. Those authors conducted a series of experiments on an aluminum plate, and the results clearly indicated the high potential of coatings with a high lignin content as anti-corrosive agents. There are also several literature reports that describe the direct use of lignin as an agent reducing the amount of mixing water for the production of cement mixtures [15,16] or as a plasticizing agent [17,18].

The continuous progress of civilization leads to increased demand for building materials, among which concrete is currently the most widespread. It is characterized by low production costs, durability and ease of adaptation. One of the possible technological advances regarding modern concrete is the use of functional mineral admixtures. Admixtures replace a part of the cement and have a positive effect on the properties of the concrete. The most commonly used mineral admixtures include fly ash and slag [19]. It has been shown that fly ash improves the water resistance and compressive strength of cement [20,21,22]. Another frequently used admixture is limestone. The authors of several reports [19,23,24] conducted a broad analysis in which they assessed, among other things, the influence of limestone on the porosity of mortars, obtaining similar results in independent studies. It was found that although a higher limestone content does not negatively affect the pore structure in cement mortar; it has a positive effect on its mechanical strength.

Other common admixtures for cement mortars, which increase their compressive strength and enable control of the setting time, include alumina [25,26,27,28,29], silica (as an admixture which leads to higher initial strength and affects mortar workability [25,29,30]), and titanium dioxide [31]. 

In recent years, numerous attempts have been made to develop functional inorganic-organic hybrid materials with improved mechanical parameters, thermal properties, biocompatibility or biodegradability, for example to improve rubber products [32,33] or to reduce the emission of harmful compounds [34,35].

Following this trend, the authors prepared functional hybrid materials, using lignin and/or lignosulfonate as the organic part and alumina as the inorganic component. The most important aspect of the research was the use of the designed systems as admixtures for cement mortars, which made it possible to achieve greater plasticity of the system while maintaining or increasing the mechanical strength of cementitious composites. It should be noted that there are no previous publications related to the use of the above-mentioned systems as admixtures for cement mortars.

## 2. Results and Discussion

### 2.1. Characteristics of Inorganic-Organic Hybrid Materials and Original Components

#### 2.1.1. FTIR Spectroscopy

**Figure 1:**
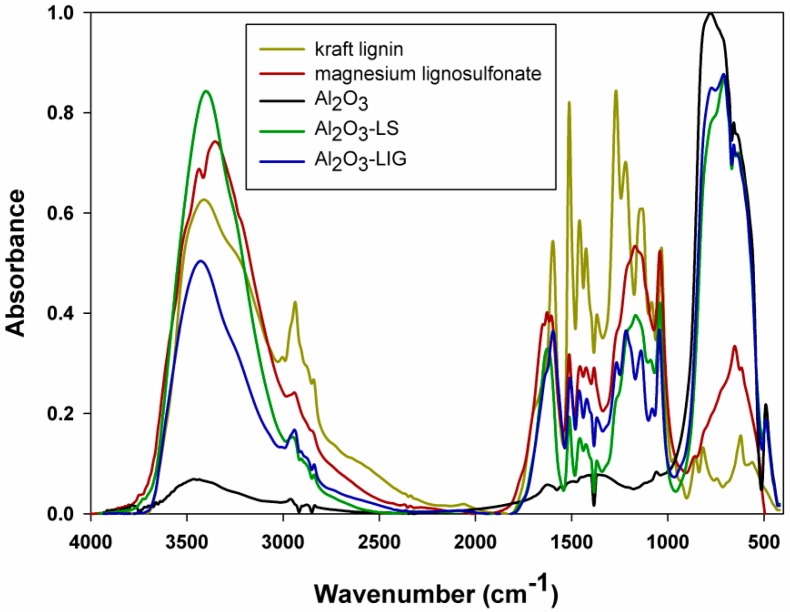
Fourier transform infrared spectroscopy (FTIR) spectra of Al_2_O_3_, lignin, lignosulfonate and inorganic-organic hybrid materials.

Analysis of Fourier transform infrared spectroscopy (FTIR) spectra of the biopolymers used was important for assessing the effectiveness of the formation of inorganic-organic hybrid materials (see Figure 1). On the spectrum for pure kraft lignin, the following bands can be observed: O–H stretching bands in a range of 3600–3200 cm^−1^, and C–H stretching vibrations in ranges of 2965–2915 cm^−1^ (CH_3_ + CH_2_) and 2855–2840 cm^−1^ (O–CH_3_). The broader band in the 1715–1560 cm^−1^ range results from the imposition of successively unbound and bound tensile vibrations of the carbonyl group C=O, while the wavenumbers 1608 cm^−1^ and subsequently 1505 cm^−1^ and 1460 cm^−1^ are associated with the C–C stretching vibrations of the aromatic backbone. In the FTIR spectral analysis, lignin bands with maximum intensity at wavenumbers 1370 cm^−1^, 1270 cm^−1^ and 1220 cm^−1^ are also important, and correspond to the stretching vibrations of C–O, C–O(H) + C–O(Ar) phenolic groups as well as ether bonds.

The stretching vibration band at wavenumber 1045 cm^−1^ also indicates the presence of C–O–C ether linkages. The last group of significant bands characteristic for lignin includes bands in the deformation plane δip Ar C–H (1140 cm^−1^) and out-of-plane δop Ar C–H (bands with wavenumbers below 1000 cm^−1^) [5,9,10,11].

The spectrum obtained for magnesium lignosulfonate is also presented in Figure 1. Similarly, to unmodified lignin, there are many characteristic signals present in the spectrum of this biopolymer, which are associated with the complex structure of the compound and the presence of a significant number of functional groups in its structure. The characteristic, intense band between 3600 cm^−1^ and 3200 cm^−1^ results from stretching vibrations of O–H moieties. The signal with a maximum at wavenumber 2940 cm^−1^ is generated by stretching vibrations of C–H groups (CH_3_ and CH_2_), as reported in [36]. The signal attributed to vibrations of CHx groups in the dactyloscopic range, at wavenumber ~650 cm^−1^, should also be highlighted. The intense band with a maximum at 1705 cm^−1^ originates from stretching vibrations of aldehyde and ketone groups. Three distinct signals at wavenumbers 1605 cm^−1^, 1515 cm^−1^ and 1420 cm^−1^ are generated by oscillations of carbon-carbon bonds in the aromatic ring, as reported in [37]. The strands characteristic for Ar–H bonds occurring at a frequency of ~3030 cm^−1^ are shielded by a strong band of hydroxyl groups. The low-intensity band visible at a frequency of ~820 cm^−1^ originates from the deformation vibrations of non-planar Ar–H bonds. Additionally, the signals at 1160 cm^−1^ and 1125 cm^−1^ are generated, respectively by the asymmetric and symmetric stretching vibrations of (Ar) C–O–C moieties.

In turn, on the spectrum shown in Figure 1, obtained for pure alumina, the presence of a wide band in the range 3600–3200 cm^−1^ is associated with stretching vibrations of hydroxyl groups, which correspond to physically adsorbed water on the surface of the hygroscopic oxide. This is confirmed by the band with a maximum at 1620 cm^−1^, associated with bending vibrations of O–H groups. The pure alumina may contain, apart from *α*-Al_2_O_3_, other allotropic forms such as *β*, which is looser in structure and can contain some Al–OH bonds (which is in accordance with the manufacturer’s specifications). As a result, a small band emerges at 1035 cm^−1^, attributed to symmetrical Al–OH bending vibrations. The last group includes bands with maxima at 750 cm^−1^, 693 cm^−1^, 564 cm^−1^, and 493 cm^−1^, which are attributed to vibrations of Al–O groups, in which aluminum ions occupy both tetrahedral and octahedral sites [7,38].

The effectiveness of the preparation of Al_2_O_3_-lignin and Al_2_O_3_-lignosulfonate hybrid materials using the mechanical method was confirmed by FTIR spectroscopy (see Figure 1). As can be observed, individual bands originating from the unmodified components are visible on the spectra of the final hybrid systems. In fact, the applied method of preparation enabled the effective combination of individual components by means of weak hydrogen bonding interactions, which is evidenced primarily by small shifts in the absorption maxima of the bands, especially for hydroxyl groups. This clearly confirms the successful preparation of inorganic-organic hybrid systems classified as class I. A mechanism for the linking of the inorganic part (alumina) and lignin was proposed in our previous publication [7].

#### 2.1.2. TGA Analysis

**Figure 2:**
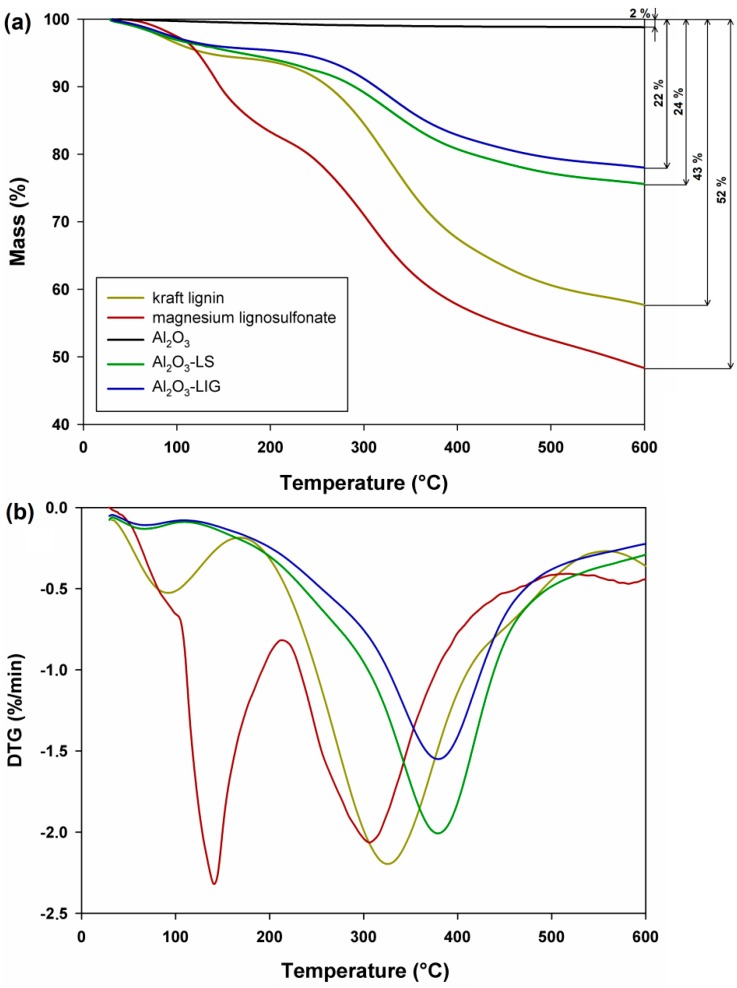
Thermogravimetric (TGA) (**a**) and DTG (**b**) curves of original components and inorganic-organic hybrid materials.

Determination of the thermal stability of the unmodified components and of the obtained inorganic-organic hybrid materials was an important aspect of their characterization. Thermogravimetric (TGA) and derivative thermogravimetric (DTG) curves of all analyzed products are presented in Figure 2.

The TGA curve for alumina indicates a low 2% loss of sample mass, which is caused by the loss of water physically bound to the surface of the material. This result confirms one of the many advantages of the inorganic precursor used, and confirms its high thermal stability over a wide temperature range [7]. The TGA curves for the biopolymers used (kraft lignin and magnesium lignosulfonate) show sample mass losses of 43% and 52% respectively, which indicates the limited thermal stability of the tested products. For both biopolymers, the first stage of mass loss (in a temperature range of ~25–220 °C) is mainly associated with the local elimination of water bound on the surface of the materials. The second stage, which entailed a much higher mass loss in a temperature range of 220–600 °C, is associated with the complex thermal decomposition of the compound, resulting in partial elimination of lignin fragments, which is related to fragmentation of the molecule due to unclear and uncontrolled reactions. These observations are confirmed by previously published papers [5,7,8,11].

In turn, the obtained Al_2_O_3_-lignin and Al_2_O_3_-lignosulfonate hybrid materials are characterized by a relatively high thermal stability, especially in the initial, most important temperature range (~200–250 °C). The TGA curves indicate unequivocally that the use of an inorganic component improved the thermal stability parameters, which has been confirmed many times, especially in the characterization of silica-lignin hybrid materials [5,39]. The mass loss of hybrid materials with lignin and lignosulfonate in the analyzed temperature range was equal to 22% and 24% respectively (see Figure 2). The favorable results obtained at this stage of the research show that hybrid materials containing polymers of natural origin can be successfully used as a new generation of environmentally friendly and relatively cheap admixtures for cement composites, especially those exposed to high temperatures.

#### 2.1.3. Dispersive-Morphological Properties

**Figure 3:**
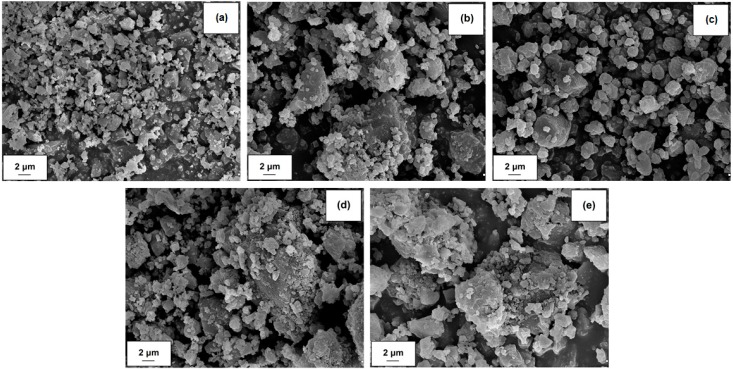
SEM images of alumina (**a**), lignosulfonate (**b**), lignin (**c**), Al_2_O_3_-lignosulfonate hybrid material (**d**) and Al_2_O_3_-lignin hybrid material (**e**).

The research also included evaluation of the dispersion and morphology of the components and hybrid materials used. The results of the tests are presented in Table 1 (particle sizes determined using a Zetasizer Nano ZS and Mastersizer 2000) and Figure 3 (scanning electron microscope images).

The tested biopolymers were characterized by the presence of irregular particles that tend to form larger agglomerates (see Figure 3). According to the particle size analysis, carried out using the Zetasizer Nano ZS apparatus, the biopolymers have relatively small particles in their microstructure, in the nanometric range (91–106 nm for lignin, 59–142 nm for lignosulfonate). However, the volume contribution of these particles is insignificant, due to the presence of larger structures, up to 5560 nm, the maximum size which can be measured using the apparatus. This information is supplemented by data obtained using a Mastersizer 2000 analyzer, which confirmed the presence of numerous larger agglomerate structures. On the basis of the results, the average particle size was estimated at 6.8 μm for lignin and 7.0 μm for lignosulfonate (see Table 1). Alumina exhibited a more homogeneous morphological character, with a particle size range of 342–2670 nm (Zetasizer Nano ZS), and an average size of 1.8 μm, as determined using the Mastersizer 2000. This was confirmed by the SEM image in Figure 3a.

As expected, the obtained hybrid materials had larger average particle sizes: 7.1 μm and 7.4 μm respectively for Al_2_O_3_-lignin and Al_2_O_3_-lignosulfonate (see Table 1). The presence of relatively larger particles in the analyzed hybrid materials was also confirmed using the Zetasizer Nano ZS. Additionally, based on the SEM images (Figure 3d,e), it can be concluded that smaller alumina particles surrounded the larger biopolymer particles and formed weak hydrogen bonding interactions, as confirmed by FTIR analysis (see Figure 1).

### 2.2. Analysis of the Properties of Cement Mortars

#### 2.2.1. Investigation of the Degree of Compactability of Cement Mortars

The produced hybrid materials were used as admixtures in cement composites, along with original components (for comparative purposes). Determination of the consistency (degree of liquidity) of the cement mortar by means of a slump test was a very important part of the research. The consistency of the mixture is a very important parameter, which should be selected depending on the method and time of transportation of the concrete mixture, the method of concreting, the shape of the element and the placement of reinforcement.

**Figure 4:**
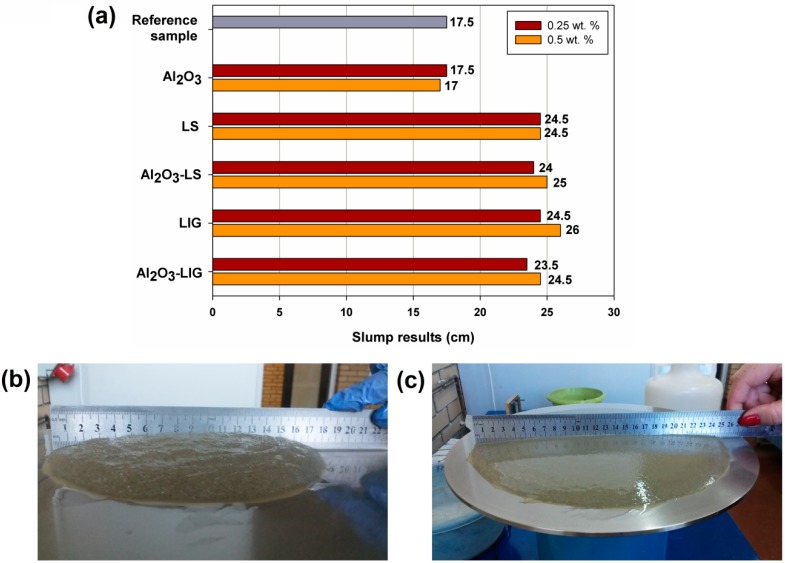
Slump test results for the analyzed samples (**a**) and digital photos of the slump for the reference sample (**b**) and the mixture containing 0.5 wt.% admixture of alumina-lignin hybrid material (**c**).

A standard test was carried out, which involved determination of the size of the slump of fresh mortar on the flow table. The obtained sizes are summarized in Figure 4a. Additionally, digital images of the tested mixtures are presented in Figure 4b,c.

The reference sample in the form of cement mortar without any admixtures produced a slump test result of 17.5 cm. The addition of 0.25 and 0.5 wt.% of Al_2_O_3_ admixture did not alter this parameter. Only the addition of biopolymers—both lignin (LIG) and magnesium lignosulfonate (LS)—resulted in an increase in the slump result (to 24.5–26.0 cm). The quantity of admixture did not significantly affect the value. The hybrid materials used in the tests as admixtures for the mortar resulted in a final increase of the slump result (23.5–25.0 cm, depending on the quantities of mortar and biopolymer used). The results show clearly that the addition of biopolymers or of hybrid systems containing biopolymers makes it possible to increase the final slump result of cement mortar, indicating that they act as plasticizers, as has also been confirmed by other authors [15,16,17,18].

#### 2.2.2. Determination of Compressive Strength of Cement Mortars

It is well known that cement-based matrices are inherently brittle, which means that they exhibit high compressive strength and low flexural and tensile strength. In common cement-based materials like mortar or concrete the values of flexural or tensile strength average about 10% of compressive strength and amount to 7–8 MPa. The admixtures proposed in this article, especially those that are organic-based, may have an adverse effect on the porous microstructure, reducing in particular the compressive strength of the material. The impact of these additives on the tensile and flexural properties is negligible, and therefore in this article only the more significant compressive strength parameter is considered.

**Figure 5:**
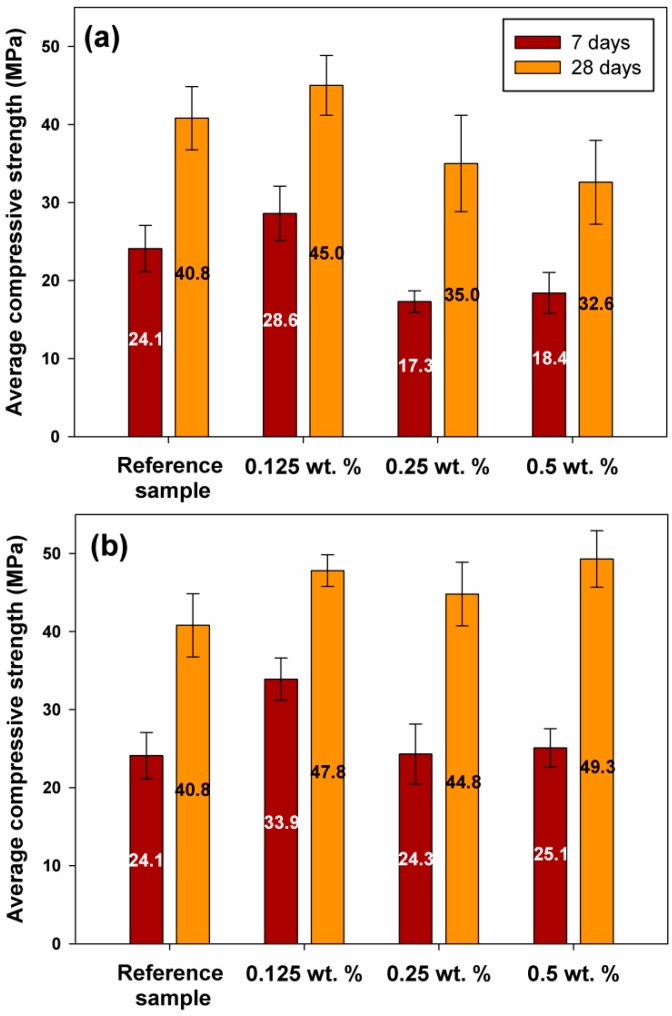
Compressive strength results for samples containing different quantities of lignosulfonate (**a**) and lignin (**b**).

Tests were conducted to determine the compressive strength of the obtained cement mortars. The results of the tests after 7 and 28 days of curing are presented in Figure 5a for samples with lignosulfonate admixture, and in Figure 5b for samples with lignin.

Analysis of the influence of lignosulfonate admixture on the strength properties of the samples showed that an increase in the content of this biopolymer in mortar results in a decrease in the average compressive load, which leads to faster destruction of samples. The best results, in comparison with the unmodified mixture, were obtained with a content of 0.125% lignosulfonate in the mortar: after 7 days a strength of 28.6 MPa was attained (compared with 24.1 MPa for the reference mixture), and after 28 days the value was 45.0 MPa (40.8 MPa for the reference mixture). Analysis of lignin admixtures in cement mortar indicated a significant increase in compressive strength in the case of a 0.125% lignin admixture after 7 days (33.9 MPa); however, increasing the lignin content results in a slight decrease in the initial strength, reaching 25.1 MPa for 0.5% biopolymer admixture (see Figure 5b). The values for the 28-day samples are considerably different. Comparable results, at ~48–49 MPa, were obtained for both the lowest (0.125%) and the highest (0.5%) biopolymer content in the mortar. In addition, it should be noted that the highest value after 28 days (49.3 MPa) was obtained in the sample containing 0.5% lignin, despite its relatively low strength after 7 days. Other authors have previously reported an increase in compressive strength for samples containing biopolymers [16,18].

**Figure 6:**
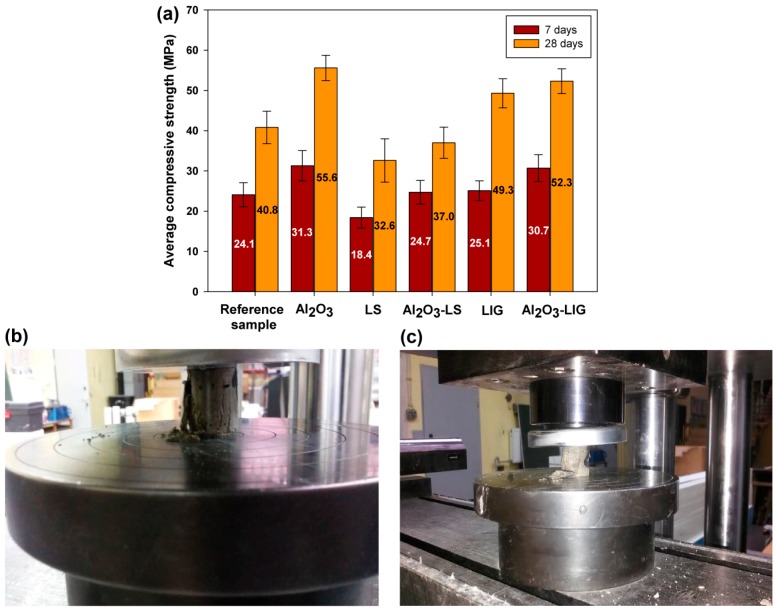
Compressive strength test results for samples containing 0.5 wt.% admixture after 7 and 28 days (**a**) and photos obtained during compressive strength tests for alumina-lignosulfonate (**b**) and alumina-lignin (**c**) hybrid materials.

It was concluded from the results of the tests that the use of a lignin admixture leads to higher values of compressive strength (compared with lignosulfonate). In addition, the highest lignin content, 0.5%, led to the product with the highest compressive strength. Based on this result, the authors conducted further tests on mortar with 0.5% admixtures. These studies involved both Al_2_O_3_-lignosulfonate and Al_2_O_3_-lignin hybrid materials, as well as an inorganic component in the form of alumina. The results of the tests are presented in Figure 6a. In addition, digital images of samples after compressive strength tests are presented in Figure 6b,c—after the maximum compressive strength is reached, the sample is destroyed. 

The analysis of mortar samples with an admixture of pure alumina indicated that the inorganic additive significantly improves the initial mortar strength both after 7 days (31.3 MPa) and 28 days of curing (55.6 MPa), compared with the mixture without additives (reference sample). This clearly indicates that the addition of an inorganic component has a significant influence on the strength of cement composites, as has also been confirmed by other authors [26,40]. As expected, good strength properties were also obtained in the case of the Al_2_O_3_-lignin admixture: high values, 30.7 MPa and 52.3 MPa, were achieved for initial strength and after 28 days of curing respectively. This finding, in addition to the improvement of the plasticizing properties obtained by the addition of lignin, makes this system suitable for further application tests with ready-made cement composites used on a larger scale in construction, which form the basis of research currently being conducted. Unfortunately, the hybrid system with lignosulfonate did not lead to improvement of the strength properties of the final mixtures. The higher compressive strength of lignin-containing hybrid systems may result from the lower tendency of that biopolymer to cause aeration of cement mixtures, in comparison to lignosulfonate [41].

#### 2.2.3. SEM Analysis of Cement Mortars

**Figure 7:**
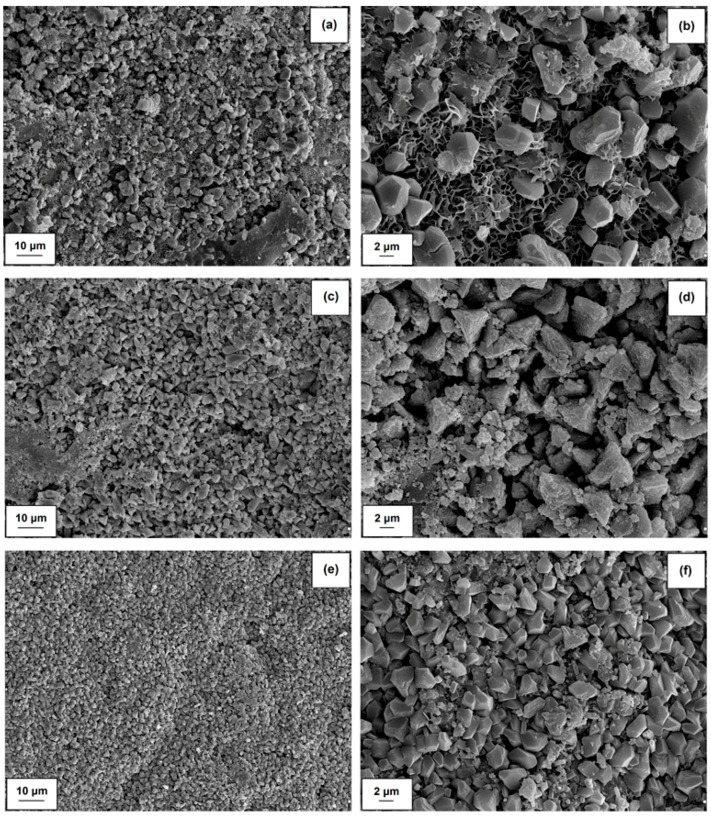
SEM images of samples after compressive strength tests for cement mortar (**a**,**b**), Al_2_O_3_-LS (**c**,**d**) and Al_2_O_3_-LIG (**e**,**f**) hybrid materials.

SEM images were prepared to evaluate the microstructural nature of unmodified cement mortar and mixtures with admixtures of hybrid materials containing alumina and lignin or lignosulfonate. The images are presented in Figure 7. In the SEM image for cement composite without any admixture (see Figure 7a,b) the grains of cement and the way in which they are cross-linked with the aggregate are clearly visible. In the subsequent SEM images of mixtures containing admixtures of the Al_2_O_3_-lignosulfonate (Figure 7c,d) or Al_2_O_3_-lignin (Figure 7e,f) hybrid system, macromolecular biofilms are clearly visible, with a multi-layered agglomerate with smaller alumina particles evenly embedded on the surface. In case of both hybrid admixtures, the analyzed products have a relatively homogeneous microstructure. A slight deformation of air bubbles is observed only in the case of the admixture of alumina-lignosulfonate hybrid material, which results from aeration of the cement mixture and negatively influences its strength properties, as demonstrated in Section 3.2.

## 3. Materials and Methods 

### 3.1. Materials

The following raw materials were used in the research: Portland CEM I 42.5R cement (Górażdże Cement S.A., Górażdże, Poland), containing Portland clinker (95%) as the main component and binding time regulator (up to 5%); alumina (Imerys Fused Minerals Villach GmbH, Villach, Austria); kraft lignin with an average molecular weight of 10,000 (Sigma Aldrich, Steinheim am Albuch, Germany); magnesium lignosulfonate (Vianplast 55, Biotech Lignosulfonate Handels GmbH, St. Valentin, Austria); and standard quartz sand (Kwarcmix, Tomaszów Mazowiecki, Poland), with φ approx. < 1 mm, designed for laboratory tests of cement strength.

### 3.2. Preparation of Alumina-Lignin and Alumina-Lignosulfonate Hybrid Materials

To obtain alumina-lignin and alumina-lignosulfonate hybrid materials with a 1:1 weight ratio, appropriate amounts of the biopolymers and inorganic precursor were weighed and then placed in an RM100 mortar grinder (Retsch GmbH, Haan, Germany) and subjected to grinding for 1 h to fragment and mix the ingredients. Then, the product was ground in a Pulverisette 6 Classic Line ball mill (Fritsch GmbH, Idar-Oberstein, Germany) for another hour. The vessel with the homogenized materials was placed concentrically on the rotary base of a planetary ball mill, in which the direction of rotation of the base is opposite to the direction of rotation of the vessel, with a speed ratio of 1:–2. The movement of the balls inside the vessel results from the Coriolis force. Different speed rates between the balls and the vessel lead to friction and impact forces, which generate high dynamic energy. The interaction of these two phenomena leads to a very high degree of fragmentation of the ground material. The mill operated at intervals, with a change of rotation direction every 15 min. To obtain appropriate homogeneity of the final material, grinding was continued for 2 h. To prevent overheating of the material as a result of continuous grinding, the mill switched off automatically for 5 min every 30 min, after which it resumed operation. Immediately after grinding, the alumina-lignin and alumina-lignosulfonate hybrid materials were sieved through an 80 μm sieve.

### 3.3. Characterization of Inorganic-Organic Hybrid Materials

The obtained hybrid materials and initial components were subjected to physicochemical and dispersive-morphological analysis.

The correctness and efficiency of the synthesis of alumina-lignin and alumina-lignosulfonate hybrid materials were confirmed using Fourier transform infrared spectroscopy (FTIR) in the 4000–450 cm^−1^ range. The spectra were obtained using a Vertex 70 spectrometer (Bruker Optik GmbH, Ettlingen, Germany). The analyzed materials were tested in the form of a pellet containing a mixture of anhydrous KBr (approx. 0.1 g) and 1 mg of the test substance. To prepare the tablets, the mixture was pressed in a special steel ring at a pressure of approximately 10 MPa under vacuum.

Thermal stability analyses were carried out using a Jupiter STA 449F3 analyzer (Netzsch GmbH, Selb, Germany), using the thermogravimetric method (TGA). A sample of appropriate mass was heated in a temperature range of 30–600 °C, with a step of 10 °C/min, under a nitrogen atmosphere. The measurement was carried out using a TGA-DTA adapter. The obtained thermogram showed the dependence between the sample mass and temperature as the latter increased over time.

Particle size analysis was carried out using a Zetasizer Nano ZS apparatus (Malvern Instruments Ltd., Malvern, UK), which enables the measurement of particle sizes in the range 0.6–6000 nm, based on the non-invasive backscattering (NIBS) technique. The particle sizes in the range 0.2–2000 μm were also measured using a Mastersizer 2000 analyzer (Malvern Instruments Ltd., Malvern, UK), based on laser diffraction.

The surface morphology, shape and size of individual particles were examined using scanning electron microscopy (SEM). An EVO40 microscope (Zeiss, Jena, Germany) was used for the study. In addition to the hybrid material and the initial components, samples of cement mortars from the compressive strength tests of cylinders were also analyzed, which enabled the natural cracks of the products to be shown on the images.

### 3.4. Preparation of Cement Composites

To obtain cement composites, the following components were weighed: 150 g of CEM I Portland cement, 270 g of sand with φ < 1.0 mm, 70 mL of water, and a given admixture (hybrid system or pure components) in a quantity of 0.25% or 0.5% by cement weight. The aggregate and cement were placed directly in the mixer, while the introduced compound was initially dispersed in water and then poured into the mixer. The prepared ingredients were subjected to vacuum mixing in a Twister series automatic mixer (Renfert GmbH, Hilzingen, Germany) for approximately 2 min at 450 rpm. The prepared composite was subjected to a flow table test to determine the degree of compactability, and then placed in cylindrical containers to obtain samples for strength tests.

### 3.5. Determination of the Consistency of Fresh Cement Mortars

The tests included determination of the slump diameter of mortar samples on a flow table, in accordance with the PN-EN 1015-3 standard. A flow table, a truncated cone-shaped mold and a compactor were used to conduct the determination. The following procedure was employed to determine the consistency of mortar: (i) before the measurement, the plate surface was wetted; (ii) the mold with the cap was placed in the center of the disk; (iii) the tested mortar was introduced into the mold in two layers—each layer was compacted by striking 10 times with a compactor; (iv) after compacting the second layer, the cap was removed, and the excess mortar was cut with a knife and smoothed to the edge of the mold; (v) 10 seconds after compacting, the mold was raised vertically and the mortar sample was shaken by rotating a special crank at a rate of 1 revolution per second; (vi) immediately after the shocks, two perpendicular diameters of the spilled mortar mix (cm) were measured with an accuracy of 1 mm.

### 3.6. Determination of Compressive Strength

Strength tests of the samples were carried out using the SATEC™ series testing machine (Instron^®^, Norwood, MA, USA). This is a static machine, designed for testing samples under compression. The tested samples were of a size of 20 mm × 20 mm, and were cylindrical in shape. The test was adopted from the PN-EN 196-1: 2006 standard. It consisted of placing the cylinders in a special insert provided with square compressive plates made of hardened steel. The plates pressed the central part of the test sample. The load was increased evenly at a speed of 2.4 ± 0.2 kN/s until the sample was destroyed. 

## 4. Conclusions

In this study, Al_2_O_3_-magnesium lignosulfonate and Al_2_O_3_-lignin hybrid materials were designed as functional admixtures for cement mortars. The effectiveness of the synthesis of inorganic-organic systems using mechanical grinding of ingredients was confirmed on the basis of spectra obtained using Fourier transform infrared spectroscopy. It was concluded that there are weak hydrogen bonding interactions between the components, which was confirmed by small shifts of the absorption maxima of appropriate functional groups, particularly hydroxyl groups. Thus, it was possible to obtain appropriate class I hybrid materials. In addition, they were shown to have relatively good thermal stability and a homogeneous microstructural character. 

An important aspect of the research was the use of the obtained hybrid materials and untreated components as admixtures for cement mortars. In the tests it was confirmed that the untreated alumina does not change the plasticity of the mixture and has a positive effect on the compressive strength, in addition to being the most homogeneous material, with the highest thermal stability. The addition of biopolymers (both lignin and lignosulfonate) increased the plasticity of the mortar cement composites by changing their strength properties. In turn, the introduction of functional hybrid materials to cement mortars resulted in a change in the compounds’ plasticity, while the compressive strength was maintained (Al_2_O_3_-lignosulfonate) or even improved (Al_2_O_3_-lignin). Thus, it is justified to use such a hybrid, which on the one hand improves the plastic properties of the mixture (due to the presence of a biopolymer), and on the other hand leads to an improvement in the mechanical properties (mainly due to the use of an inorganic component). It appears justified to continue the development of this line of research, particularly in relation to the application of the above-mentioned admixtures in ready-made construction products, which is currently being implemented.

## Figures and Tables

**Table 1:** Dispersive properties of lignin, lignosulfonate, alumina and inorganic-organic hybrid materials.

Sample Name	Dispersive Properties				
	Particle Size Distribution from Zetasizer Nano ZS (nm)	Particle Diameter from Mastersizer 2000 (μm)			
		d(0.1) ^1^	d(0.5) ^2^	d(0.9) ^3^	D[4.3] ^4^
Lignin	91–106, 712–1110, 2670–5560	2.0	5.4	8.3	6.8
Lignosulfonate	59–142, 955–1484, 2670–5560	2.2	5.7	8.4	7.0
Al_2_O_3_	342–2670	0.8	1.6	2.4	1.8
Al_2_O_3_-lignin (1:1 wt./wt.)	122–615, 1484–5560	2.3	5.5	8.6	7.1
Al_2_O_3_-lignosulfonate (1:1 wt./wt.)	164–955, 1720–5560	2.6	5.8	8.8	7.4

^1^ d(0.1) – 10% of the volume distribution is below this diameter value; ^2^ d(0.5) – 50% of the volume distribution is below this diameter value; ^3^ d(0.9) – 90% of the volume distribution is below this diameter value; ^4^ D[4.3] – average particle size in the examined system.
